# Editorial: “Homeostasis and Allostasis of Thyroid Function”

**DOI:** 10.3389/fendo.2018.00287

**Published:** 2018-06-05

**Authors:** Johannes W. Dietrich, John E. M. Midgley, Rudolf Hoermann

**Affiliations:** ^1^Medical Department 1, Endocrinology and Diabetology, Bergmannsheil University Hospitals, Ruhr University of Bochum, Bochum, North Rhine-Westphalia, Germany; ^2^Ruhr Centre of Rare Diseases (CeSER), Ruhr University of Bochum, Bochum, North Rhine-Westphalia, Germany; ^3^Ruhr Centre of Rare Diseases (CeSER), Witten/Herdecke University, Bochum, North Rhine-Westphalia, Germany; ^4^North Lakes Clinical, Ilkley, United Kingdom; ^5^Private Consultancy, Research and Development, Yandina, QLD, Australia

**Keywords:** thyroid hormones, thyronamines, homeostasis, allostasis, feedback regulation, hysteresis, TACITUS syndrome, syndrome T

**Editorial on the Research Topic**

**Homeostasis and Allostasis of Thyroid Function**

## Current Challenges in Thyroidology

A basic understanding of thyroid control involving pituitary thyrotropin (TSH) has become a cornerstone for the contemporary diagnosis of thyroid disorders. However, long-held simplistic interpretations of the classical feedback concept fall short of the elusive goal of a universally applicable and reliable diagnostic test. Diagnostic ambiguities may arise from the dynamic nature of thyroid homeostasis. Concentrations of TSH and T3 are governed by circadian (1) and, additionally for TSH, ultradian rhythms (2). Plasticity of the hypothalamic–pituitary–thyroid axis in form of adaptive responses may promote misdiagnosis, especially in pregnancy and critical illness (3, 4). Diagnosis of subclinical dysfunction is also dependent on the mode of statistical analysis (5–9).

Consequently, the clinical care of thyroid patients faces major challenges, foremost ill-defined reference ranges for TSH and thyroid hormones (THs), and persistently poor quality of life in a substantial subset of treated hypothyroid patients (10). Divergent criteria by guidelines for defining thyroid disease and guiding therapeutic intervention have further added to the confusion. It remains unclear, if patients with subclinical hypothyroidism benefit from treatment and which are sensible targets of substitution therapy (11, 12).

By addressing predictive adaptation, the rather new theory of allostasis complements the established concept of homeostasis. In situations of strain and stress, allostasis ensures *stability through change* by modifying setpoints and parameters of feedback control (13–15). Despite being a basically beneficial reaction allostasis may also expose the organism to a new kind of strain referred to as allostatic load, which may result in even life-threatening diseases.

This research topic focusing on homeostasis and—still understudied—allostasis of thyroid function was initiated with the goal that deeper physiological insights in pituitary–thyroid feedback control may aid in solving the aforementioned problems. A series of articles summarizes the state of current scientific knowledge, and delivers new perspectives, as significant progress has been made in that regard.

## Thyroid Homeostasis—Unexpected Complexities in a Classic Endocrine Feedback Loop

A review article by the editors (Hoermann et al.) provides an overview of homeostatic mechanisms in the light of recent research. The classical “short feedback” structure (*Astwood-Hoskins loop*) (16) is now complemented by additional motifs, an “ultrashort” autocrine loop, where TSH inhibits its own secretion, and a TSH-T3 shunt relaying stimulation from pituitary to intrathyroidal step-up deiodinases. Although documented for decades on a biochemical level (17, 18), the clinical importance of the TSH-T3 shunt has only recently been recognized (19–23).

Newly identified non-classical processing structures add to the complexity of the control system. They explain both pulsatile thyrotropin release and significant deviations from a log-linear relationship between FT4 and TSH concentrations [Hoermann et al.; (24–26)]. In onset hypothyroidism, rising TSH concentrations stimulate T3 formation (22), thereby maintaining thyroid signaling and unburdening the thyroid from T4 synthesis (Hoermann et al.).

A balancing concept for TSH, FT4, and FT3 is introduced under the term *relational stability* [Hoermann et al.; (22)]. Importantly, it is lacking in athyreotic patients and suspended when treatment with L-T4 reduces TSH concentration—an important argument against universal L-T4 substitution in subclinical hypothyroidism.

The novel clinical concepts feed back to theory. Berberich et al. describe an expanded physiology-based mathematical model of thyroid homeostasis that incorporates the rediscovered TSH-T3 shunt. This model extends a rich tradition of related “parametrically isomorphic” models (27–35), demonstrating that circadian variations of FT3 concentrations are well explained by TSH action and shedding a fresh light on the evolution of subclinical thyroid diseases (Berberich et al.).

Interpretation of thyroid function tests can be severely affected by homeostatic time constants resulting in hysteresis effects (36), as reviewed by Leow, extending implications to antithyroid treatment and LT4 substitution.

## Technological Advancements and Novel Diagnostic Tools

Although sensitive for primary hypothyroidism, TSH measurement has low specificity and is unable to detect dysfunctions of central origin. Isolated TSH measurements may be misleading in certain physiological (37) and allostatic conditions (38), including non-thyroidal illness (39).

In a short perspective article, we summarize methodological principles and clinical trial results (Dietrich et al.) for novel diagnostic approaches based on mathematical modeling, such as functional thyroid reserve capacity and step-up deiodinase activity. These calculated parameters deliver estimates for “hidden” structure parameters of thyroid homeostasis and provide early indicators of thyroid failure. Reconstructing the individual equilibrium point (the so-called *set point*) of thyroid homeostasis is facilitated by new tools and may prove useful as a personal target for L-T4 dosage titration (40, 41). Mathematical modeling can further improve interpretation of L-T4 absorption tests (42).

## The Enigmatic Role of Non-Classical TH

The world of THs is composed of more than T4 and T3. Today, we know 27 metabolites derived from the thyronine skeleton, some of them being hormonally active [Hoermann et al.; (43)]. Thyronamines have received special attention, binding to trace amine-associated receptors (44) and acting as functional antagonists of iodothyronines (45, 46).

Glossmann et al. critically appraise suggested pharmacological uses of 3-monoiodothyronamine (3-T1AM), e.g., for therapy of stroke or in long-lasting space flights. Based on its pleiotropic effects they question if 3-T1AM can be a safe cryogenic drug. Some of the inconsistencies in reported serum concentrations may result from plasma protein binding, potential role of gut microbiota in the formation of thyronamines from iodothyronines or conversion of 3-T1AM to 3-iodothyroacetic acid (3-TA1), a possible major mediator of thyronaminergic signaling (47).

## Hypothalamus–Pituitary–Thyroid Axis—an Open and Dynamic System

The traditional view of pituitary–thyroid feedback control holding T4 plasma concentration constant close to a fixed set point (48) has been challenged by variable concentrations of TSH and THs in certain physiological situations beyond thyroid disease (38, 49–55). Thyroid allostasis delivers a unified theory for a plethora of adaptive processes spanning from fetal life, pregnancy, starvation, exercise, obesity, aging, and general severe illness to psychiatric disorders. In strain and stress, type 1 and type 2 allostasis affect thyroid function in different ways, creating each distinctly recognizable patterns (Chatzitomaris et al.).

## Prospectus

Deeper insights in the physiology of thyroid function and its homeostatic control warrant a rethinking of diagnostic practice. The old paradigm employing TSH in the center of diagnostic work-up has to be replaced by a relational concept, where TSH is interlocked with FT4 and FT3, and multivariable distributions represent homeostatic equilibria (9, 30). This new approach allows for personalized interpretation of thyroid function and understands physiological influences as constituents of homeostatic/allostatic control modes (Hoermann et al.).

## Author Contributions

JD, JM, and RH wrote some of the papers in this Research Topic and participated as guest editors for manuscripts, where they were not coauthors themselves. All authors listed have made a substantial, direct, and intellectual contribution to this editorial and approved it for publication.

## Conflict of Interest Statement

JD received funding and personal fees by Sanofi-Henning, Hexal AG, Bristol-Myers Squibb, and Pfizer and is co-owner of the intellectual property rights for the patent “System and Method for Deriving Parameters for Homeostatic Feedback Control of an Individual” (Singapore Institute for Clinical Sciences, Biomedical Sciences Institutes, Application Number 201208940-5, WIPO number WO/2014/088516). All other authors declare that there is no conflict of interest that could be perceived as prejudicing the impartiality of the research reported.
